# A Multiplex Real-Time Reverse Transcription Polymerase Chain Reaction Assay With Enhanced Capacity to Detect Vesicular Stomatitis Viral Lineages of Central American Origin

**DOI:** 10.3389/fvets.2021.783198

**Published:** 2021-12-20

**Authors:** Kate Hole, Charles Nfon, Luis L. Rodriguez, Lauro Velazquez-Salinas

**Affiliations:** ^1^National Centre for Foreign Animal Disease, Canadian Food Inspection Agency, Winnipeg, MB, Canada; ^2^Foreign Animal Disease Research Unit, Plum Island Animal Disease Center, United States Department of Agriculture-Agricultural Research Service, Greenport, NY, United States

**Keywords:** vesicular stomatitis, real time PCR, diagnostic, genetic diversity, epidemic lineage

## Abstract

Vesicular stomatitis virus (VSV) causes a disease in susceptible livestock that is clinically indistinguishable from foot-and-mouth disease. Rapid testing is therefore critical to identify VSV and rule out FMD. We previously developed and validated a multiplex real-time reverse transcription polymerase chain reaction assay (mRRT-PCR) for detection of both VS New Jersey virus (VSNJV) and VS Indiana virus (VSIV). However, it was subsequently apparent that this assay failed to detect some VSNJV isolates in Mexico, especially in genetic group II, lineage 2.1. In order to enhance the sensitivity of the mRRT-PCR for VSNJV, parts of the assay were redesigned and revalidated using new and improved PCR chemistries. The redesign markedly improved the assay by increasing the VSNJV detection sensitivity of lineage 2.1 and thereby allowing detection of all VSNJV clades. The new assay showed an increased capability to detect VSNJV. Specifically, the new mRRT-PCR detected VSNJV in 100% (87/87) of samples from Mexico in 2006-2007 compared to 74% for the previous mRRT-PCR. Furthermore, the analytical sensitivity of the new mRRT-PCR was enhanced for VSNJV. Importantly, the modified assay had the same sensitivity and specificity for VSIV as the previously published assay. Our results highlight the challenges the large genetic variability of VSV pose for virus detection by mRRT-PCR and show the importance of frequent re-evaluation and validation of diagnostic assays for VSV to ensure high sensitivity and specificity.

## Introduction

Vesicular stomatitis virus (VSV) is an arbovirus and prototype of the *Rhabdovirus* viral family and *vesiculovirus* genus from which vesicular stomatitis Indiana virus (VSIV) and vesicular stomatitis New Jersey virus (VSNJV) constitute the main serotypes (1). VSV has a single-stranded negative-sense RNA genome structured in five different genes, encoding five structural proteins: nucleoprotein (N), phosphoprotein (P), matrix (M), glycoprotein (G) and the large RNA-dependent polymerase (L) (2).

The genetic diversity of VSNJV has been associated with at least six different phylogenetic clades, which are directly linked to the geographical regions where these viruses are typically circulating (3). The homology among VSNJV has been calculated between 79.56 and 85.16% and 91.04 and 94.66% at the nucleotide and amino acid levels, respectively (3).

In southern Mexico, where VSV is endemic, clinical cases are recorded on an annual basis (4). These VSV endemic zones are colonized by multiple lineages belonging to North American Clade I (3). Most VSV outbreaks recorded in the United States have been linked to endemic ancestors from these regions (4–6).

Between 2005 and 2011 two interesting epidemiological events occurred in Mexico. The emergence of a highly virulent epidemic lineage 1.1 (7), which affected central and northern Mexico was reported, and was subsequently isolated in the US in 2012 (4). Concurrently, an incursion of multiple lineages belonging to the Central America clade II were described for the first time in Mexico (4). Although these lineages were initially found to produce clinical infections in livestock in southern Mexico, by 2011 clinical cases associated with these lineages were detected in central Mexico. This implies that VSV may spread from Central America into the US via Mexico.

VSV clinical manifestations, such as epithelial lesions, resemble the ones produced by foot and mouth disease virus (FMDV), one of the most economically devastating livestock diseases worldwide (8). As such, the differential diagnosis of VSV is performed on a regular basis. Real-time reverse transcription polymerase chain reaction (rRT-PCR) is one of the quickest diagnostic and most valuable tests for this purpose (9–12). In this context, we consider it imperative to have continuous validation of this diagnostic mRRT-PCR assay to ensure the accuracy and reliability over time, especially given the genetic heterogeneity of VSV and the possibility for sequence mutations within the target gene.

We initially published the development and the validation of a mRRT-PCR that targets a specific region of the L gene; an assay capable of detecting and serotyping VSIV and VSNJV strains from field samples in a single reaction (13). This was followed by a reevaluation and subsequent validation using representative strains of different VSNJV genetic groups (3), to extend its range of detection associated with genetic groups II, IV, V, and VI (10).

Herein, we are presenting the results of the second redesign and validation of our mRRT-PCR assay to enhance the detection of VSNJV associated with the genetic group II. We aimed to evaluate the performance of our mRRT-PCR assay to detect samples from both the Central American lineage 2.1 recently introduced in Mexico and the epidemic lineage 1.1. The results of this study are discussed in terms of the importance of maintaining a continuous validation program of the mRRT-PCR protocols used in the detection of VSV. This highlights the relevance of molecular epidemiology studies using multiple methods to promote the detection of new lineages of this virus and the consequent improvement of routine diagnostic tools.

## Methods

### Sample Collection

**Figure 1:**
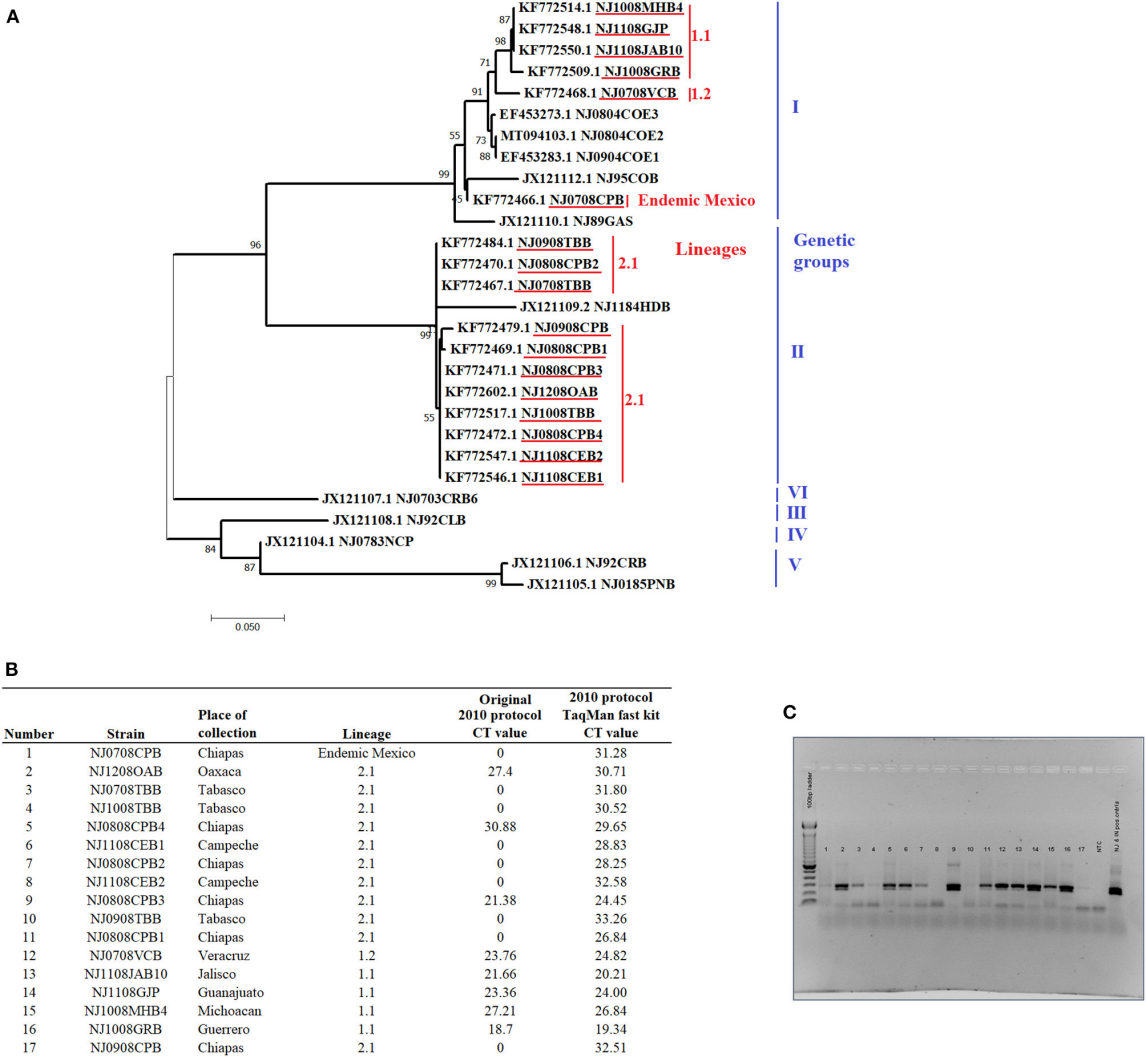
General information and analysis of the 17 epithelial samples obtained from naturally infected livestock with VSNJV in Mexico during 2008. **(A)** Phylogenetic analysis reconstructed by maximum likelihood method representing the genetic relationship of VSNJV in the 17 tissue suspensions used for the validation of this study (highlighted in red). The analysis was enhanced using VSNJV isolates representing different genetic groups of this serotype. **(B)** Results of the comparison of the 2010 protocol using different master mix kits. **(C)** Gel visualization of the amplicons from the mRRT-PCR reactions using the original 2010 protocol.

A total of 17 tissue suspensions from epithelial samples collected during 2008 from naturally infected cows in eight different states of Mexico were obtained from the Mexico-United States Commission for Prevention of Foot-and-Mouth Disease and other Animal Exotic Diseases (CPA). These samples represent the genetic diversity of VSNJV in Mexico from 2005 to 2011 and include VSNJV associated with genetic clades I including a sample representing the endemic lineages in Mexico, as well as samples from lineages 1.1 and 1.2 (the most recent common endemic ancestor of lineage 1.1) (*n* = 6) and lineage 2.1 (*n* = 11) (Figure 1A). These samples were from a molecular epidemiology study conducted in Mexico and were previously determined to be positive for VSNJV by double antibody sandwich ELISA (DAS ELISA) for VSV antigen detection, viral isolation, conventional RT-PCR, and sanger sequencing (4). Specific details about these samples are presented in Figure 1.

### RNA Extraction and mRRT-PCR Performance

Viral RNA was extracted using the MagMax™-96 viral RNA isolation kit (AM1836, Applied Biosystems) and a KingFisher automated extraction system (ThermoFisher Scientific) following the manufacturer's instructions. The presence of VSNJV by mRRT-PCR was evaluated using an Applied Biosystems 7500 real-time PCR platform. Initially, samples were evaluated using the 2010 protocol and conditions (10). Briefly, master mix preparation was carried out using the Platinum Quantitative RT-PCR Thermoscript One-Step System kit (Applied Biosystems #11731015), in a final volume of 25 ul. Amplification conditions were as follows: one cycle of reverse transcription for 30 min at 50°C, followed by 1 min at 95°C, and 45 cycles of 95°C for 15 s, 54°C for 30 s, and 72°C for 1 min (Data collection). Additionally, to evaluate the potential effect of the master mix kit in the reaction, we performed this protocol using the TaqMan™ Fast Virus 1-Step Master Mix kit (Applied Biosystems #4444434) in a final volume of 25 ul. Amplification conditions for this kit are as follows: For reverse transcription 1 cycle of 5 min at 50°C followed by 95°C for 20 s, and 45 cycles of 95°C for 15 s and 60°C for 45 s (Data collection).

### Primers, Probes and Amplification Conditions Developed for the 2021 Protocol

The set of primers and probes included in the 2021 protocol for the VSIV component, produce a predicted amplicon size of 227 bp. The nucleotide locations in the L gene are based on the VSIV strain Mudd-Summers (GenBank: ). Primers are described as: 7230F 5'-TGATACAGTACAATTATTTTGGGAC-3' (7230-7254 nucleotides), and 7456R 5'-GAGACTTTCTGTTACGGGATCTGG-3' (7456-7433). The probe IN 22 was labeled at the 5'-end with the reporter dye VIC and MGB (minor groove binder) was incorporated at the 3'-end. 5'-VIC-ATGATGCATGATCCTGCTCTTC-MGB-3' (7274-7295). The primers and probes included in the VSNJV component produce a predicted amplicon size of 266 bp. The nucleotide locations in the gene L are based on the VSNJV strain NJ0612NME6 (GenBank: MG552609.1) Primers were described as:7230F-1: 5'-TGATTCAATATAATTATTTTGGGAC-3' (7133-7157), 7230F-2: 5'-TGATTCAATATAATTACTTTGGAAC-3 (7133-7154) and REV2: 5'-AGGCTCAGAGGCATGTTCAT-3' (7398-7379). Probes were labeled at the 5'end with the reporter dye FAM and MGB was incorporated at the 3'end. These were identified as: M1 5'-FAM-TTTATGCATGATCCCGCAATACG-MGB-3' (7177-7199), M2 5'-FAM-TTTATGCATGACCCTGCCATAAG-MGB-3' (7177-7199), and short probe 5'-FAM-TTGCACACCAGAACAT-MGB-3' (7237-7252). Details about the development of the 2021 protocol are presented in the results and discussion sections.

The master mix for the reaction was made with the TaqMan™ Fast Virus 1-Step Master Mix kit in a final volume of 25 ul. Final 1× mix included: RNase-Free Water 12.25 μl, 4× TaqMan Fast Virus 1-step Master Mix 6.25 μl, Forward primer mix (7230F +7230F-1 +7230F-2: 0.2 μM each) 0.5 μl, reverse primer mix (7456R + REV2: 0.2/0.8μM, respectively) 0.5 μl, and probe mix (short + M1+ M2 + IN 22: 0.1, 0.05, 0.05, 0.1μM, respectively) 0.5 μl, and RNA template 5 μl. Amplification conditions were as described above for the TaqMan™ Fast Virus 1-Step Master Mix kit. More information about this is available in the Supplementary File 1.

### Amplification Efficiency, Analytical Sensitivity and Diagnostic Specificity

The determination of amplification efficiency and analytical sensitivity of the new protocol were performed as previously described (14). Several 10-fold dilutions of a known titer of either VSNJV or VSIV reference strains (Ogden and San Juan, respectively) were prepared and triplicates of each dilution were assessed by mRRT-PCR to ascertain the limit of detection, defined as the last dilution where all repetitions were positive in all three replicates. To assess the accuracy of the results, this experiment was performed three times.

For the diagnostic specificity a total of 80 negative cattle epithelial tissues were evaluated, as well as different viruses associated with the production of vesicular lesions in livestock. This analysis comprised of six FMDV strains including serotypes A, O, Asia 1, SAT 1, SAT 2 and SAT 3, two strains of swine vesicular disease virus (SVDV) and two strains of Senecavirus A (SVA).

### Phylogenetic Analysis

To evaluate the genetic relationship between the 17 viruses included in this study and the six genetic groups of VSNJV, a phylogenetic analysis was reconstructed by Maximum likelihood method under the general time reversible model, with a bootstrap analysis of 1,000 replicates to assess the accuracy of the reconstruction. Analysis was conducted on the software MEGA X (15). Alignments were conducted with Jalview version 2.11.1.3 using the Clustal W algorithm.

### Sanger Sequencing

Primers used for the purpose of sequencing were the previously described 7230F-1 and REV2 located in the gene L. PCR products were purified using QIAquick PCR purification kits (Qiagen). Sanger sequencing of these products was performed using a BigDye Terminator Cycle Sequencing kit (Applied Biosystems). Sequencing reactions were run on a 3500xL genetic analyzer (Applied Biosystems) and the raw data was analyzed using Geneious Prime version 2021.0.3 (Biomatters Ltd.).

## Results and Discussion

The results indicated that our 2010 protocol was unable to detect 8 out of the 11 samples associated with clade II lineage 2.1 and one sample associated with endemic lineages of Mexico (Figure 1B). Conversely, this protocol was able to detect all samples associated with clade I lineages 1.1 and 1.2. When PCR products were visualized by agarose gel electrophoresis, amplification products matching the expected size of VSNJV amplicon were observed in most of the mRRT-PCR negative reactions indicating failure of VSNJV detection in these samples by mRRT-PCR might be associated with mismatches in the probes. In addition, the presence of weak bands (low amount of amplicons) observed in some samples was potentially due to issues with the primers (Figure 1C). We then evaluated the potential effect of the TaqMan™ Fast Virus 1-Step Master Mix kit and the new cycling conditions on the performance of the 2010 protocol. Interestingly, the use of this new kit and amplification conditions allowed for the detection of all samples, albeit at suspicious or high Ct values and weak curves (Figure 1B), demonstrating the positive effect that the use of this kit and amplification conditions have for this protocol.

**Figure 2:**
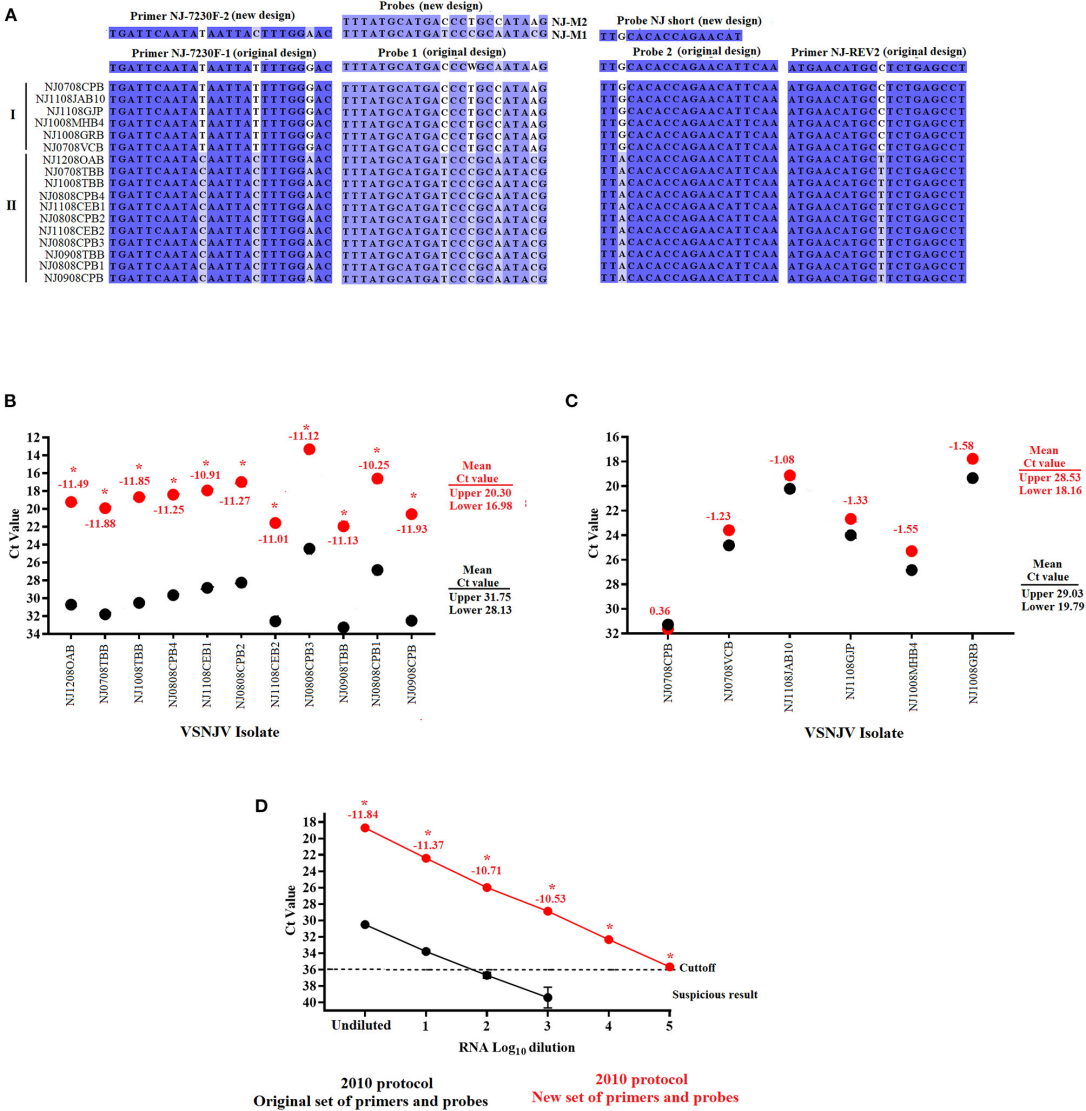
Development and validation of the new set of primers and probes for the VSVNJ component. **(A)** Detection of mutations associated with viruses from lineage 2.1 in the target region of the L gene. The sequence of new primers and probes designed to update the 2010 protocol are shown. The performance of the 2010 protocol using either the original or with a combination between some of the original and the new designed primerson **(B)** 11 samples from Central American lineage 2.1, and **(C)** 6 samples representing 1.1, 1.2 and endemic lineages from Mexico. **(D)** Comparison in the analytical sensitivity between the two versions of the 2010 using several 10-fold dilutions of RNA extraction from a representative virus of lineage 2.1.Upper and lower means were calculated using JMP PRO software with the standard error set at *p* < 0.05. Numbers under different symbols represents the differences in the Ct values between samples evaluated by the two versions of the 2010 protocol. Asterisks represent statistically significant values (*p* < 0.05) obtained by the paired *T*-test conducted in GraphPad 9.0.0; symbols represent the average of three replicates.

To assess the presence of mutations potentially causing specific mismatches between regions of the L gene and the primers and probes of this assay, Sanger sequencing was conducted using the forward and reverse primers described in our 2010 protocol (10). Consistent with the results observed in the analysis of negative reactions in the agarose gel, a total of seven mutations were present in all samples associated with the lineage 2.1 viruses in both the primers and probe regions; mismatches at forward primer (*n* = 3), probe 1 (*n* = 3) and reverse primer (*n* = 1) (Figure 2A). Though using TaqMan fast enabled the 2010 mRRT-PCR to detect VSNJV in all samples tested, we hypothesized that the mismatches in the primers and probes diminished the overall performance of the assay, especially the analytical sensitivity. Based on these results, and to improve the assay to better detect VSNJV from a Central America origin, primers and probes were modified based on the sequences obtained from the Mexico isolates and were further evaluated. Overall, we included a new forward primer (NJ-7230F-2), replaced the 2010 probe 1 with probes M1 and M2 (representing clades I and II), and substituted probe 2 with a shorter probe at the same location (Figure 2A). For the VSIV component of the assay, the only modification in relation to the 2010 protocol, was the addition of five nucleotides at the 3' end of the IN 22 probe to promote the stability of this reagent in the reaction. Blast analysis of IN 22 probe showed 100% of identity with all VSIV isolates reported in GenBank database including all isolates reported from the most recent VSIV outbreak in the USA (16).

To evaluate the effect of the newly designed primers and probes in the detection of lineage 2.1, the 2010 protocol was performed using either the original primers and probe set or a combination including some of the original assay and the newly designed primers and probes (see Methods section for exact information about this mix. Both versions were performed using the TaqMan™ Fast Virus 1-Step Master Mix kit.

Compared to the results obtained using the 2010 protocol with the original set of primers and probes, the 11 samples associated with lineage 2.1 showed a statistically significant (*p* < 0.05) decrease in the Ct values (between −10.25 and −11.88), producing a significant 3.5-4 log_10_ improvement in terms of diagnostic sensitivity for this lineage when using a combination primer and probe mix containing some of the original set and the new set (Figure 2B). Conversely, no statistically significant differences were found in the six samples associated with lineages 1.1, 1.2 and endemic Mexico (Figure 2C). Furthermore, the increase in the diagnostic sensitivity for the detection of samples from lineage 2.1 was evidenced when the performance of the 2010 protocol was compared again using either the original or a mix containing both new and old primers and probes (Both using the TaqMan™ Fast Virus 1-Step Master Mix kit) using multiple 10-fold serial dilutions of viral RNA from a sample belonging to the 2.1 lineage (NJ1008TBB) (Figure 2D). Although both assays exhibited comparable amplification efficiency R^2^ values (~0.99), the 2010 protocol using a mix between the original and new set of primers and probes appeared statistically (*p* < 0.05) 1,000 times more sensitive than the same protocol using the original set of primers and probes, showing the positive effect that the new set of primers and probes have in the detection of lineage 2.1 (Figure 2D).

Based on the results expressed above, we changed the conditions of our 2010 protocol to include a primer-probe mix containing a combination of some of the original set and the newly designed set of primers and probes. We also incorporated the TaqMan™ Fast Virus 1-Step Master Mix kit and the amplification conditions for this kit to establish the improved assay referred to as the 2021 protocol.

**Figure 3:**
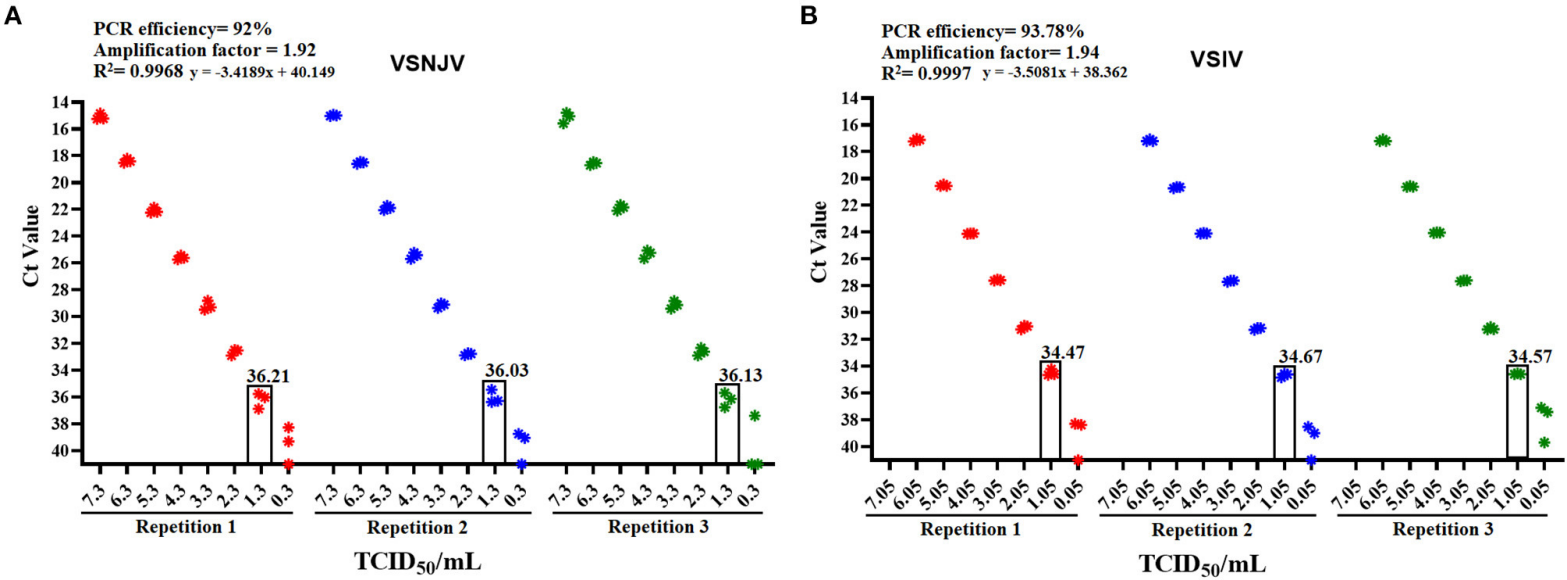
Determination of the amplification efficiency and the analytical sensitivity values of the 2021 protocol using reference strains of each serotype. Initial validation of the 2021 protocol was conducted on the **(A)** VSNJV and **(B)** VSIV components of this protocol. In each graphic are depicted values of PCR efficiency, amplification factor, and R^2^ correlation values associated with the PCR efficiency. The analytical sensitivity of each component is shown in the rectangle that corresponds to the last dilution where all three replicates were detected.

To get a better perspective of the overall performance of the 2021 protocol, a validation testing was performed. The validation analysis showed that the 2021 protocol is highly sensitive for the detection of VSNJV and VSIV serotypes (1.3 and 1.05 TCID_50_/ml, respectively), both the PCR efficiency and amplification factor parameters were found to be optimal (17) (Figures 3A,B). No positive reactions were obtained after 45 cycles of amplification when negative epithelial samples were tested, resulting in a diagnostic specificity of 100%. In addition, no cross-reactivity was observed when representative isolates of FMDV, SVDV and SVA were evaluated with this protocol. Based on these results, samples were considered positive with Ct values <36, suspicious between Cts 36-40 and negative with a Ct > 40. Furthermore, comparability testing of the 2021 protocol on additional real-time PCR machines including the CFX96 Touch (BIO RAD) and the QuantStudio 7 Pro (Applied Biosystems) gave similar results indicating the robustness of this assay across platforms.

To further validate the performance of the 2021 assay, a historical collection of VSV samples stored at the NCFAD were tested and compared to previous results. These samples included VSNJV samples from Mexico (*n* = 87) collected between 2006 and 2007, Colombia (*n* = 78), VSIV from Colombia (*n* = 74) collected between 1996 and 2002 as well as multiple VSNJV samples (*n* = 11) (representative of all six genetic groups) and VSIV (*n* = 4) representing multiple lineages from the Americas received from PIADC.

**Figure 4:**
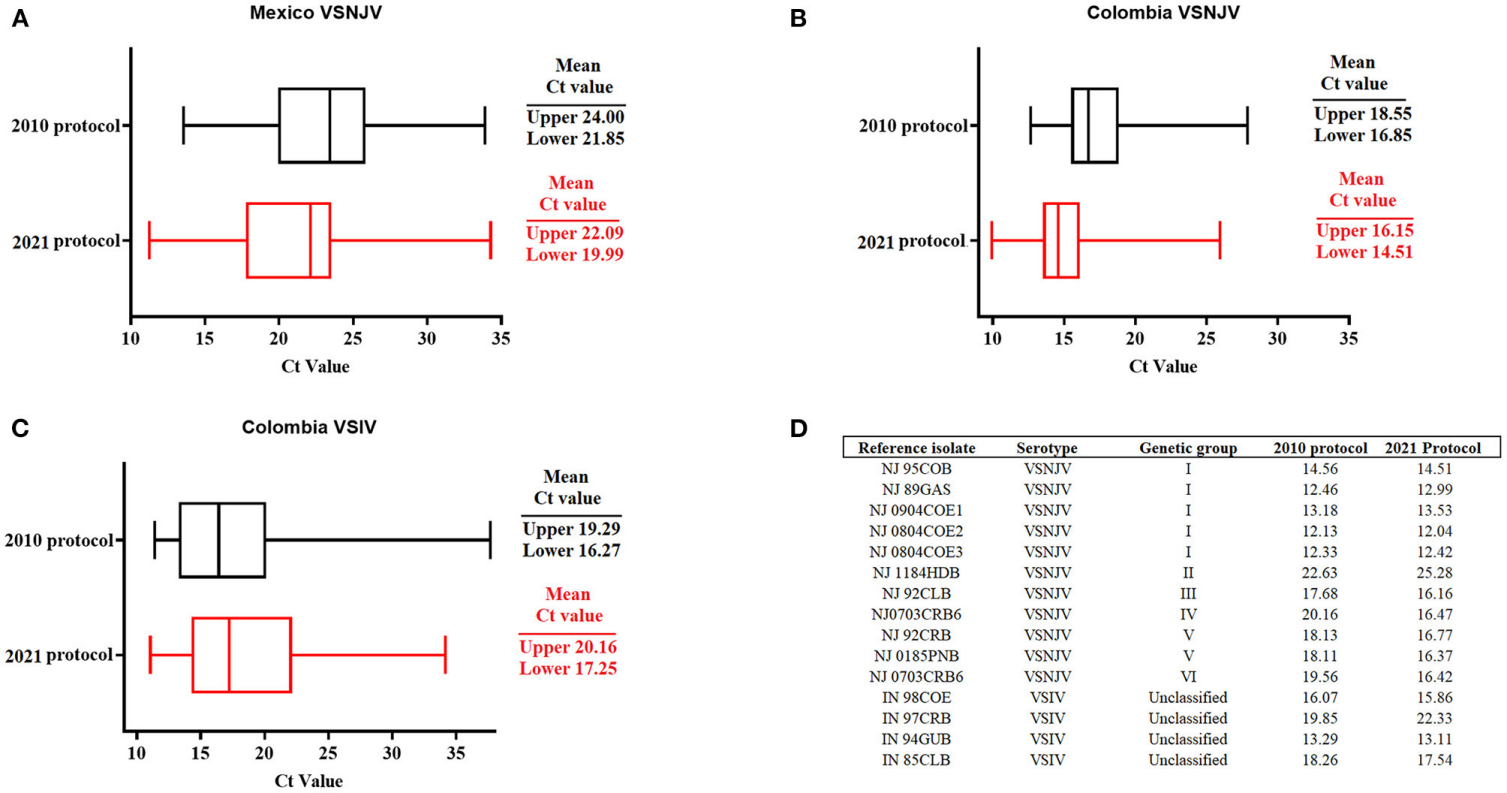
Validation of the 2021 protocol for detection of VSV. Validation was performed using a set of VSNJV field samples from **(A)** Mexico, **(B)** Colombia, and VSIV field samples from **(C)** Colombia. **(D)** Isolates representing the different genetic groups of VSNJV and VSIV. Comparisons were established using historical values obtained during the validation of the 2010 protocol. Upper and lower means of Cts were calculated using JMP PRO software with the standard error set at *p* < 0.05.

Interestingly, with the VSNJV samples from Mexico, we observed an overall slight decrease in the mean of the Ct values between the original 2010 protocol (10), and the newly established 2021 protocol (Figure 4A). Within this group, a statistically significant (*p* < 0.05) decrease in the Ct values was recorded in a total of 20 samples, showing an improvement in analytical sensitivity for these samples. The 2021 protocol was also able to detect 100% of the samples (87/87) compared with a detection rate of 74.1 % (65/87) using the 2010 protocol. This represents an increase of 25.9% in the diagnostic sensitivity for detection of lineages circulating in Mexico. Similar results were found in the overall mean Cts during the evaluation of VSNJV samples from Colombia (Figure 4B). In this context, we saw a significant (*p* < 0.05) decrease in Ct values in a total of 25 samples and an increase in the diagnostic sensitivity of 1.2% for the detection of all samples. Conversely, a slight increase in the overall mean of Ct values was seen in the 2021 protocol when compared with the 2010 protocol during the evaluation of VSIV from Colombia (Figure 4C). Furthermore, we recorded an improvement in the diagnostic sensitivity for VSIV, from 94.5% (70/74) to 100% when using the 2021 protocol.

Finally, similar to the 2010 protocol, the 2021 protocol was able to detect not only all reference VSNJV isolates representing all genetic groups of this serotype, but also all isolates associated with VSIV (Figure 4D). In case of VSNJV, there was a slight decrease in the Ct values for isolates representing the genetic groups III, IV, V, and VI. We observed a slight increase in the Ct value for the detection of the isolate associated with the genetic group II. VSIV isolate IN97CRB, also had an increased Ct (Figure 4D). Furthermore, low Ct values (Cts of 18-19) were obtained with the 2021 protocol, detecting viral isolates NJ0612NME6, and IN0919WYB1 associated with the 2012 VSNJV and 2019 VSIV outbreaks in the US, respectively (16, 18). Interestingly, during the revision process of this manuscript, we were able to evaluate the ability of the 2021 protocol to detect a group of five representative isolates (NJ0911CPB7, NJ0911CPB1, NJ1011TBP3, NJ0911TBB3, NJ0911CPB10) of the central American lineage 2.2 circulating in Mexico during 2011 (4), confirming the ability of this protocol to detect additional VSNJV lineages from central America (Ct values ranged between 17 and 19).

Overall, our data shows that the updating the primers and probes for VSV mRRT-PCR and using TaqMan™ Fast Virus 1-Step Master Mix kit substantially improves the detection of the Central America VSNJV lineage 2.1. This new assay is a vast improvement over previous ones for the detection of VSNJV group II which has high genetic variability. As lineage 2.1 has the potential to cause outbreaks in northern Mexico and the southern USA (4), it could be readily identified using this assay thereby making it an important diagnostic tool. Currently, studies are being conducted in Mexico to determine the molecular epidemiology of this lineage.

We think that the combination of two main factors contributed to the improved performance of the 2021 protocol. The most important changes were the sequence of the primers and probes that were redesigned in the new assay to accommodate mutations in the mRRT-PCR target on the L gene. The other important change was the TaqMan™ Fast Virus 1-Step Master Mix kit in place of the Platinum Quantitative RT-PCR Thermoscript One-Step System kit. The effect of this variable was evidenced by the overall improvement of the 2010 protocol. However, the inclusion of the new set of primers and probes in the VSNJV component of this assay was clearly a key factor to improve the detection of the central American lineage 2.1 Regarding the improvement in the sensitivity of the VSIV component of the assay, the most likely explanation may be associated with the use of the new amplification kit and conditions. However, at this point, we can't rule out the potential positive effect that the increase in the length of probe IN 22 might have in the performance of this component of the assay.

Considering the absence of sequencing information from the historical Mexican samples (2006-2007), we cannot rule out the possibility that some of these samples were associated with central America lineages, as they were identified in Mexico in 2005 (4). This may therefore explain improvement in the diagnostic sensitivity observed with the 2021 protocol for VSNJV samples from Mexico. Along with the molecular changes to the assay and based on the information provided by the manufacturer, one of the features of the TaqMan™ Fast Virus 1-Step Master Mix kit is its ability to better handle PCR inhibitors which may explain the added improvement this chemistry also provides to the assay. This feature may be especially important considering that the main target of this protocol is the use of clinical samples mostly represented by epithelial tissues.

The results presented in this study show the difficulties involved in the diagnosis of VSV by mRRT-PCR assay due to the heterogeneity of VSNJV. Based on these findings, we consider it imperative to perform continuous molecular epidemiology studies to increase the knowledge regarding the tremendous genetic diversity associated with this virus. In this context, although we demonstrate the capability of our protocol to detect VSNJV from all genetic groups, we consider that one of the main limitations of this study is the reduced number of samples used to support the development of the new primers and probes presented herein. Thus, we consider it imperative to conduct future validations using a vast number of samples to deeply cover the genetic variability within the different genetic groups.

Furthermore, this study exposes the necessity to keep a continuous validation program in diagnostic laboratories to ensure the correct performance of VSV mRRT-PCR using recent field isolates and the evaluation of new master mix kits.

In conclusion, we consider that the 2021 protocol presented herein, represents an excellent option not only for diagnostic purposes, but also for research laboratories that perform various studies with VSV (19–21).

## Data Availability Statement

The original contributions presented in the study are included in the article/Supplementary Material, further inquiries can be directed to the corresponding author/s.

## Author Contributions

KH and LV-S conceived and designed the experiments and analyzed the data. KH performed the experiments. KH, CN, LR, and LV-S wrote the manuscript. All authors contributed to the article and approved the submitted version.

## Funding

This work was supported by the Canadian Food Inspection Agency (WIN-A-1302) and the USDA Research Service CRIS Project No. 8064-32000-059-00D.

## Conflict of Interest

The authors declare that the research was conducted in the absence of any commercial or financial relationships that could be construed as a potential conflict of interest.

## Publisher's Note

All claims expressed in this article are solely those of the authors and do not necessarily represent those of their affiliated organizations, or those of the publisher, the editors and the reviewers. Any product that may be evaluated in this article, or claim that may be made by its manufacturer, is not guaranteed or endorsed by the publisher.

## Supplementary Material

Supplementary File 1 Protocol for primer and probe mixes preparation.
