# Supplementary material for: A Multiplex Real-Time Reverse Transcription Polymerase Chain Reaction Assay With Enhanced Capacity to Detect Vesicular Stomatitis Viral Lineages of Central American Origin
Source: Front Vet Sci. 2021 Dec 20;8:783198. doi: 10.3389/fvets.2021.783198 (PMC8720762; doi:10.3389/fvets.2021.783198)
Supplement: Supplementary File 1 — Protocol for primer and probe mixes preparation. [file Table_1.pdf]

**VSV forward primer mix**	**100 µl**	**500 µl**
Rnase-Free Water	70 µl	350 µl
7230F-1 [100µM] VSNJV	10 µl	50 µl
7230F-2 [100mM] VSNJV	10 µl	50 µl
7230F [100mM] VSIV	10 µl	50 µl
Total volume	100 µl	500 µl

**VSV reverse primer mix**	**100 µl**	**500 µl**
Rnase-Free Water	50 µl	250 µl
REV2 [100µM] VSNJV	40 µl	200 µl
7456R [100mM] VSIV	10 µl	50 µl
Total volume	100 µl	500 µl

**VSV combo probe mix**	**100 µl**	**500 µl**
Rnase-Free Water	85 µl	425 µl
Short [100µM] VSNJV	5 µl	25 µl
M1 [100mM] VSNJV	2.5 µl	12.5 µl
M2 [100mM] VSNJV	2.5 µl	12.5 µl
IN 22 [100mM] VSIV	5 µl	25 µl
Total volume	100 µl	500 µl
